# Supplementary material for: Antibody Reveals Conformational Latch Controlling Herpesvirus Proteases
Source: bioRxiv. 2025 Oct 17:2025.10.17.683123. Preprint. [Version 1] doi: 10.1101/2025.10.17.683123 (PMC12632753; doi:10.1101/2025.10.17.683123)
Supplement: 1 [file NIHPP2025.10.17.683123V1-supplement-1.pdf]

bioRxiv preprint doi: https://doi.org/10.1101/2025.10.17.683123; this version posted October 17, 2025. The copyright holder for this preprint (which was not certified by peer review) is the author/funder, who has granted bioRxiv a license to display the preprint in perpetuity. It is made available under aCC-BY 4.0 International license.

Supplementary Figures

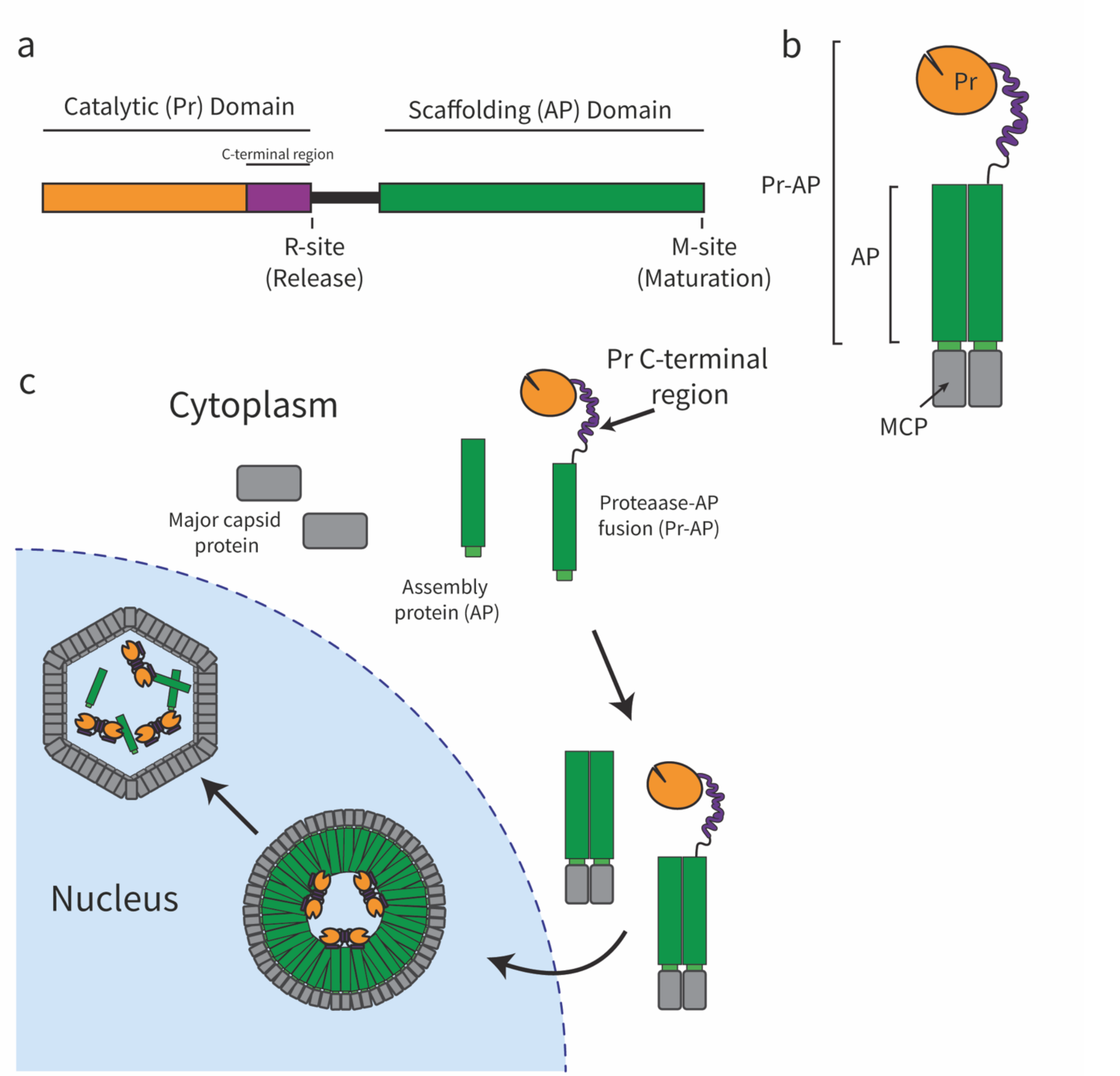

Supplementary Fig. 1: HCMV Pr gene and function (a-b) The HCMV UL80 gene. Primary components are the catalytic domain, a linker region, and the assembly protein (AP) Scaffolding domain. Proteins can either be expressed as Pr-AP or AP due to multiple open reading frames. (c) Pr-AP associates with the major capsid protein (MCP) in the cytosol. Catalytically competent Pr frees itself from AP via the R-site, and the scaffolding is cleared away via the M-site. The wild-type Pr domain contains multiple autoproteolytic sites, which is why our expression construct contains mutations (A141V, A143V, P144A and A209V) to limit autoproteolysis.

bioRxiv preprint doi: https://doi.org/10.1101/2025.10.17.683123; this version posted October 17, 2025. The copyright holder for this preprint (which was not certified by peer review) is the author/funder, who has granted bioRxiv a license to display the preprint in perpetuity. It is made available under aCC-BY 4.0 International license.

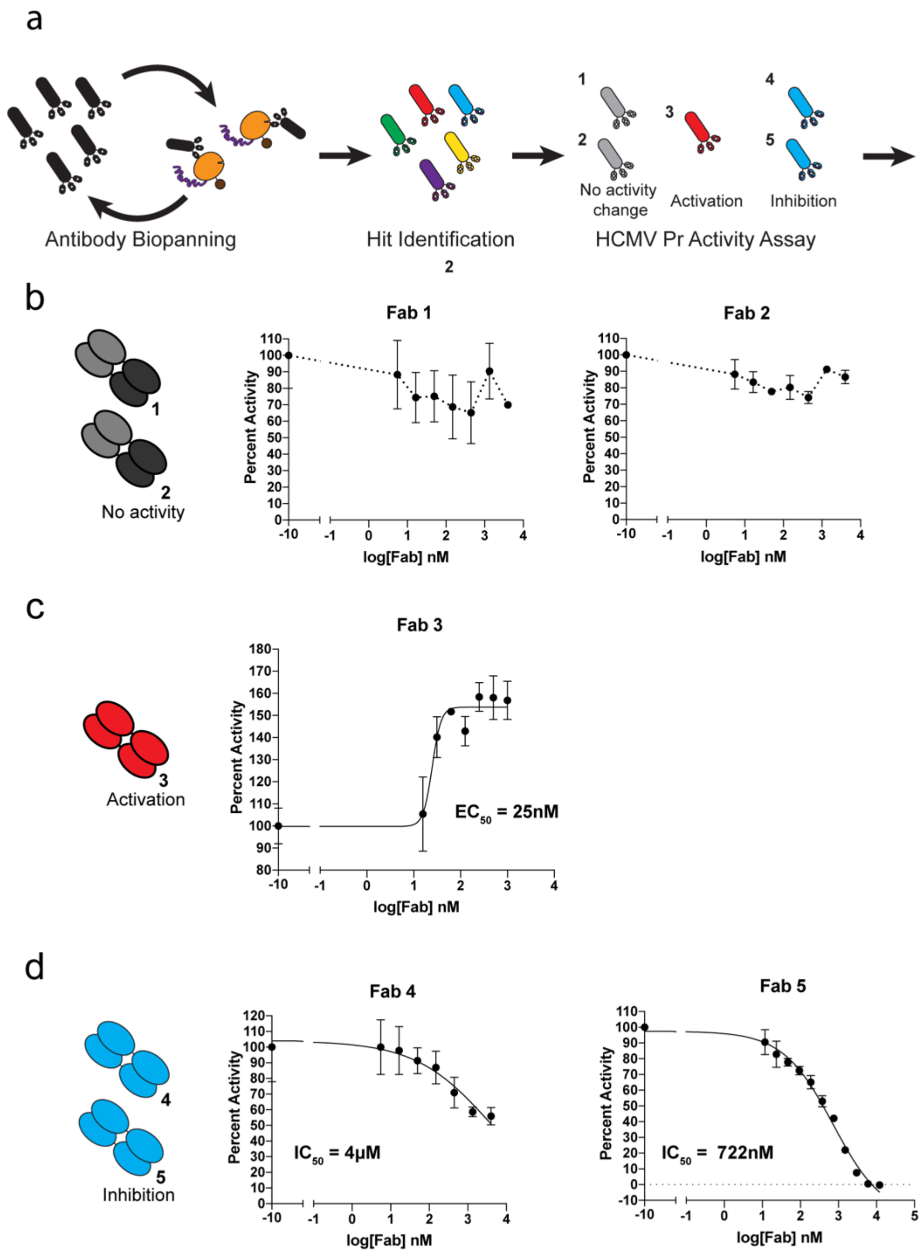

Supplementary Fig. 2: Inhibition assay with the five unique clones from HCMV Pr phage-displayed panning (a) a schematic of the panning campaign against HCMV Pr. Five hits were expressed and purified then tested for inhibition of HCMV Pr activity. (b) Fabs 1 and 2 do not affect HCMV Pr activity. (c) Fab3 has an activating effect on HCMV Pr activity. (d) Fabs 4 and 5 inhibit HCMV Pr. We chose Fab5 for deeper characterization. We did attempt to move forward with further characterization of Fab3 as well, but we were not able to purify a stable complex on SEC or SEC/MALS.

bioRxiv preprint doi: https://doi.org/10.1101/2025.10.17.683123; this version posted October 17, 2025. The copyright holder for this preprint (which was not certified by peer review) is the author/funder, who has granted bioRxiv a license to display the preprint in perpetuity. It is made available under aCC-BY 4.0 International license.

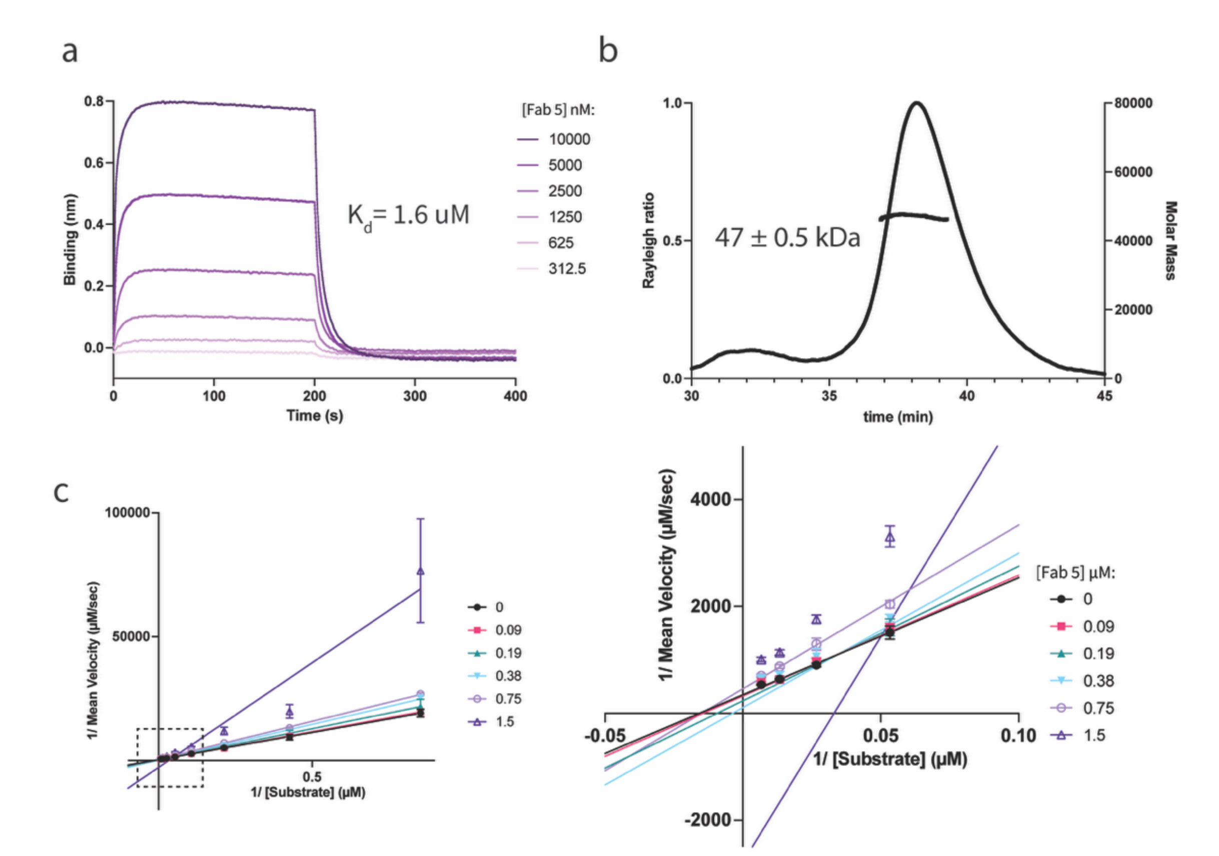

d

[Fab5] μM	K_m (μM)	V_{max} (μM/sec)
0	50.5 ± 5.1	0.00254 ± 0.0001
0.09	47 ± 8.2	0.00235 ± 0.0002
0.19	49.1 ± 7.8	0.00237 ± 0.0002
0.38	46 ± 11.7	0.00210 ± 0.0002
0.75	58.7 ± 7.5	0.00199 ± 0.0001
1.5	59.36 ± 11.8	0.00144 ± 0.0001

Supplementary Fig. 3: Fab5 BLI, SEC/MALS, and Steady-State Kinetics Assays (a) Biolayer interferometry sensogram of Fab5 binding to immobilized HCMV Pr. The dissociation constant (K_d) was determined by fitting the steady-state response to a 1:1 equilibrium binding model. (b) SEC/MALS experiment with Fab5 alone using an S200 10/300 SEC column. (c) Double-reciprocal plot of steady-state kinetics assay measuring HCMV Pr activity performed with Fab5 at various concentrations. Full data is plotted in panel 1, and a magnified view of the dotted line-boxed region is plotted in panel 2. (d) The K_m and V_{max} of HCMV Pr steady-state kinetics are provided in a table for each concentration of Fab5.

bioRxiv preprint doi: https://doi.org/10.1101/2025.10.17.683123; this version posted October 17, 2025. The copyright holder for this preprint (which was not certified by peer review) is the author/funder, who has granted bioRxiv a license to display the preprint in perpetuity. It is made available under aCC-BY 4.0 International license.

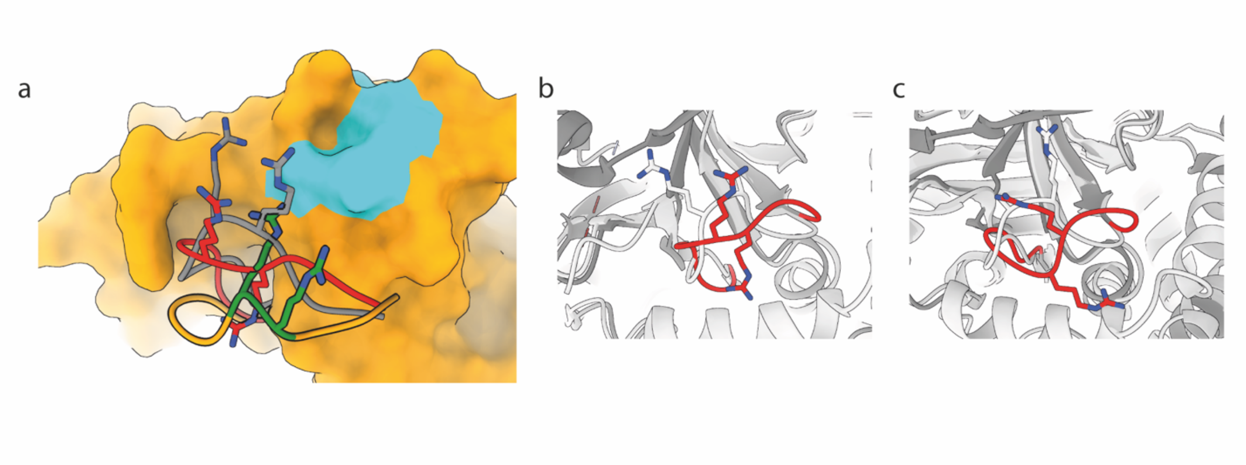

Supplementary Fig. 4: Structural comparison of the OHL (a) HCMV Pr (orange surface) with active site labeled cyan. The three loops in the foreground are OHL loops from an HCMV Pr dimer (gray, PDB:1CMV), inhibited KSHV Pr (red, PDB:3NJQ), and the Fab5-HCMV Pr complex (orange and forest green). (b-c) The biological assembly of inhibited KSHV Pr in the crystal structure PDB:3NJQ is dimeric despite the deletion of the C-terminal region. The inhibitor, which mimics helix α5 and displaces it, forms a crystal contact between 2 monomers. The OHL assumes different conformations in chains A and B (red), however both OHL conformations are more open compared to the wild-type structure (light gray, PDB:1FL1) and the highlighted arginines point away from the catalytic triad.

bioRxiv preprint doi: https://doi.org/10.1101/2025.10.17.683123; this version posted October 17, 2025. The copyright holder for this preprint (which was not certified by peer review) is the author/funder, who has granted bioRxiv a license to display the preprint in perpetuity. It is made available under aCC-BY 4.0 International license.

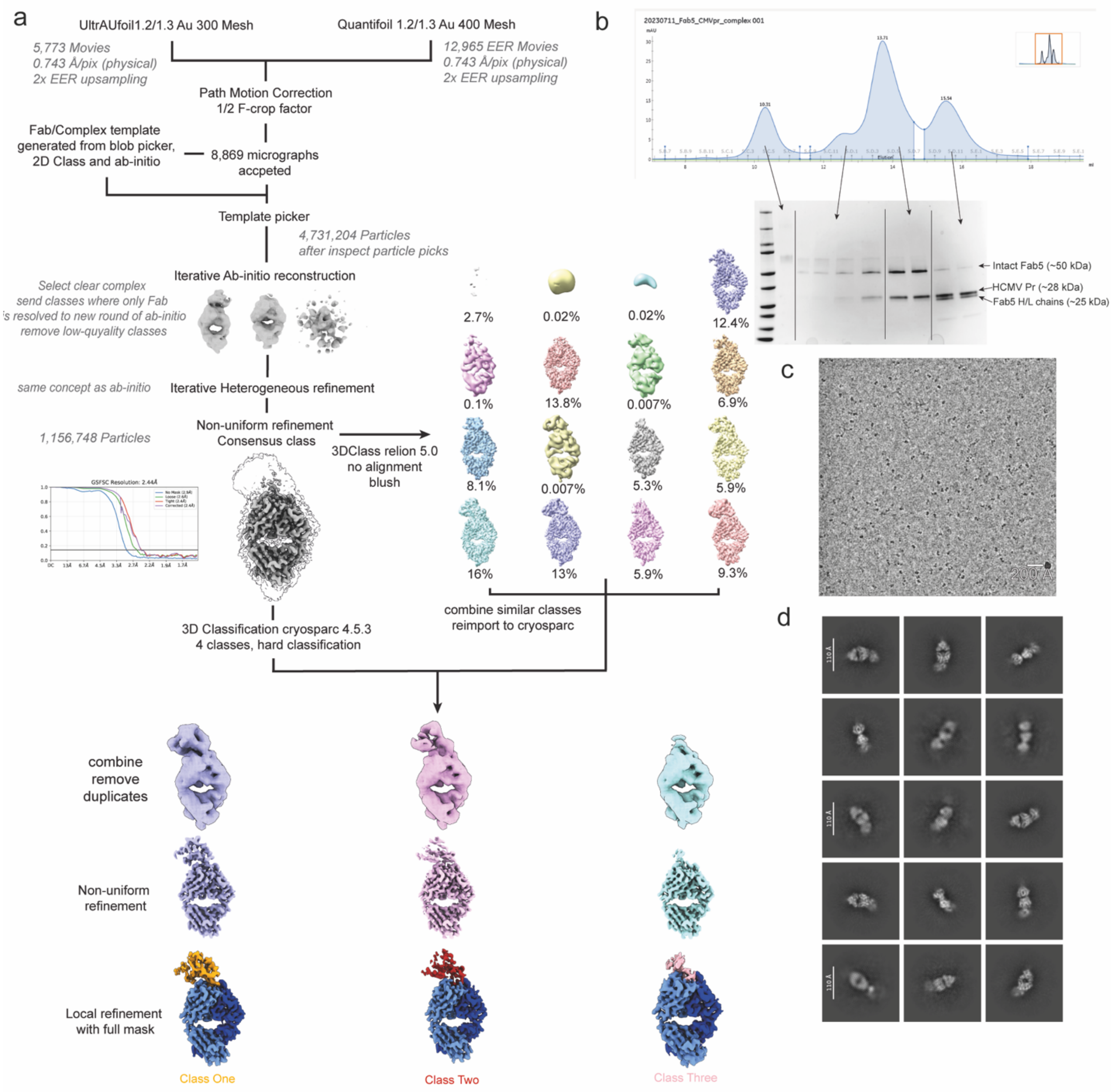

Supplementary Fig. 5: Cryo-EM Data Processing Workflow (a) Flow chart of EM processing workflow. Final volumes for classes One, Two, and Three were solved using 3D classification, non-uniform refinement, and local refinement with a full mask. All volumes were used iteratively for model building. (b) size-exclusion chromatography of Fab5/HCMV Pr complex preparation. Only the largest peak was used for cryo-EM sample. (c) Representative micrograph of Fab5/HCMV Pr complex. (d) Representative 2D classes of Fab5/HCMV Pr complex.

bioRxiv preprint doi: https://doi.org/10.1101/2025.10.17.683123; this version posted October 17, 2025. The copyright holder for this preprint (which was not certified by peer review) is the author/funder, who has granted bioRxiv a license to display the preprint in perpetuity. It is made available under aCC-BY 4.0 International license.

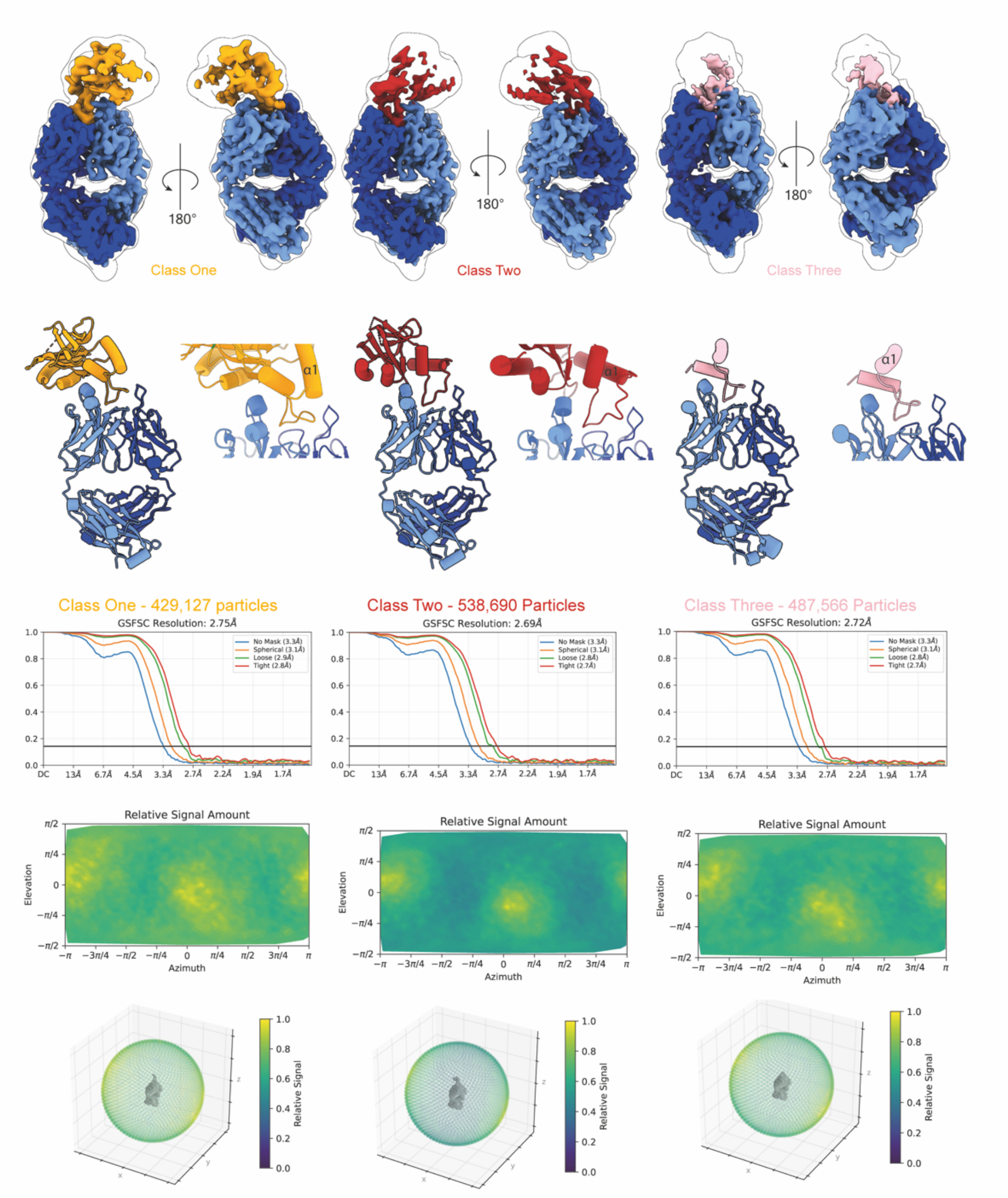

Supplementary Fig. 6: Cryo-EM Map Quality and Models For each class, two views of the map are provided with an outline of the density at a low threshold. The descending panels show the model with an inset zoom of the epitope region, then a Fourier shell correlation (FSC) curve, then orientation diagnostics where relative signal is shown as a function of viewing direction.

bioRxiv preprint doi: https://doi.org/10.1101/2025.10.17.683123; this version posted October 17, 2025. The copyright holder for this preprint (which was not certified by peer review) is the author/funder, who has granted bioRxiv a license to display the preprint in perpetuity. It is made available under aCC-BY 4.0 International license.

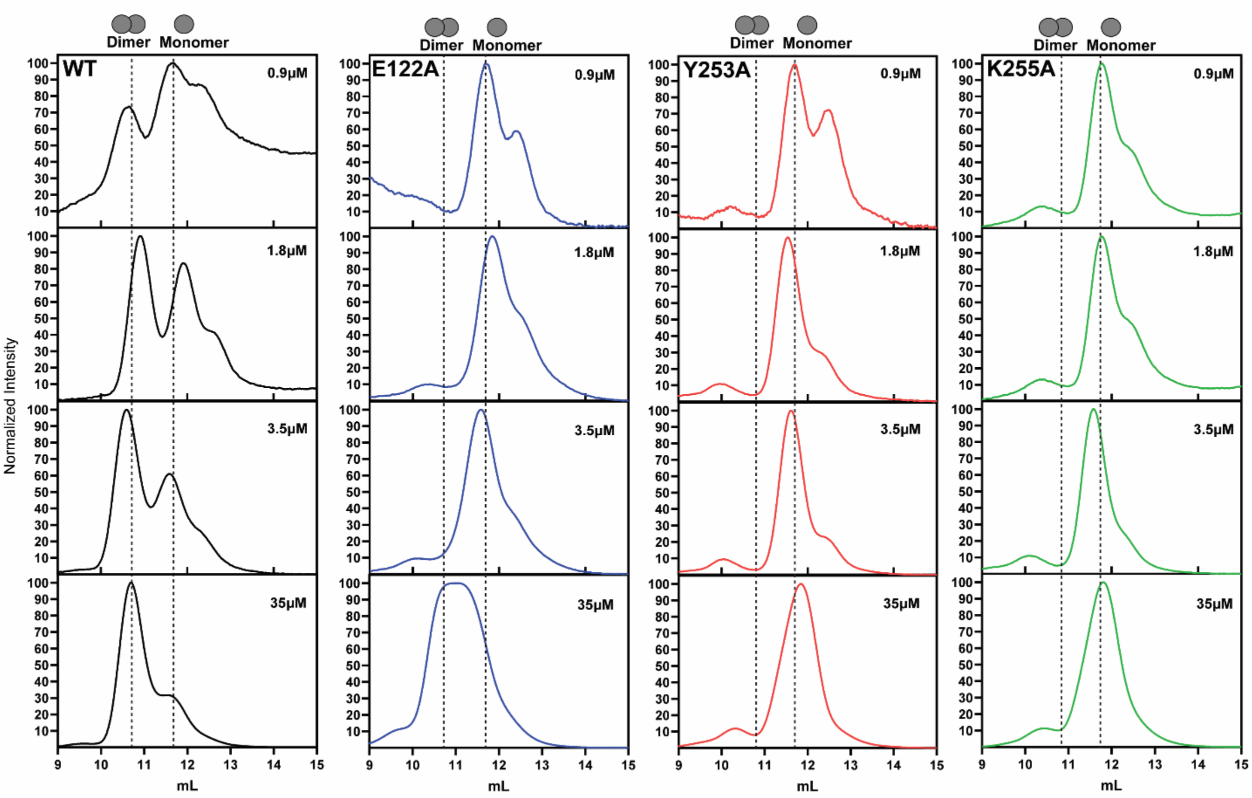

Supplementary Fig. 7: All SEC chromatograms from dimerization experiments. Peak areas were integrated, and a ratio was taken to make the percent dimer graph in Fig. 4. Results are normalized for intensity, and the x-axis has column volume. The left column is wild-type HCMV Pr, and concentration-dependent dimerization can be observed. All mutant constructs remain as monomers except for E122A, which can dimerize at the highest concentration.
